# Retrospective study for the universal applicability of the residue-based linear free energy relationship in the two-state exchange of protein molecules

**DOI:** 10.1038/s41598-022-21226-z

**Published:** 2022-10-07

**Authors:** Daisuke Fujinami, Seiichiro Hayashi, Daisuke Kohda

**Affiliations:** 1 Division of Structural Biology, Medical Institute of Bioregulation, Kyushu University, Maidashi 3-1-1, Higashi-ku, Fukuoka 812-8582 Japan; 2 Present Address: Graduate School of Integrated Pharmaceutical and Nutritional Sciences, University of Shizuoka, Yada 52-1, Suruga-ku, Shizuoka 422-8526 Japan

**Keywords:** NMR spectroscopy, Biophysical chemistry, Protein folding, Proteins, Kinetics, Molecular conformation, Thermodynamics, Perturbations

## Abstract

Multiprobe measurements, such as NMR and hydrogen exchange studies, can provide the equilibrium constant, K, and rate constants for forward and backward processes, k and k′, of the two-state structural changes of a polypeptide *on a per-residue basis*. We previously found a linear relationship between log K and log k and between log K and log k′ for the topological exchange of a 27-residue bioactive peptide. To test the general applicability of the residue-based linear free energy relationship (rbLEFR), we performed a literature search to collect residue-specific K, k, and k′ values in various exchange processes, including folding-unfolding equilibrium, coupled folding and binding of intrinsically disordered peptides, and structural fluctuations of folded proteins. The good linearity in a substantial number of the log–log plots proved that the rbLFER holds for the structural changes in a wide variety of protein-related phenomena. Among the successful cases, the hydrogen exchange study of apomyoglobin folding intermediates is particularly interesting. We found that the residues that deviated from the linear relationship corresponded to the α-helix, for which transient translocation had been identified by other experiments. Thus, the rbLFER is useful for studying the structures and energetics of the dynamic states of protein molecules.

## Introduction

The double logarithmic plot of equilibrium constants, K, and rate constants, k, is generally called REFER (rate-equilibrium free energy relationship), and the linear relationship in the REFER plot is referred to as LFER (linear free energy relationship). LFER is widely observed in two-state chemical and enzymatic reactions and is utilized to estimate the reaction rates under arbitrary conditions^1–3^. In general, the perturbations of K and k result from modifications in the structures of target compounds. For example, the data points (log K, log k) of the Hammett plot are obtained from a series of derivatives of a reactant with different substituents, and those of the Brønsted plot are obtained from a series of acids. The LFER is also seen in protein folding processes. The ϕ-value analysis is based on the perturbation to K and k by mutations of an amino acid residue in a protein molecule and provides information on the localized structure formation around the mutated position in the transition state of the protein folding^4–8^.

NMR provides local information around nuclei at an atomic resolution. We used NMR to determine the residue-specific equilibrium constants, K, and residue-specific forward and backward rate constants, k and k′, of a 27-residue peptide, nukacin ISK-1, in a two-state slow exchange^9,10^. For accurate determination of the thermodynamic and kinetic parameters, the measurement bias arising from *state-specific* differences in the R_1_ and R_2_ relaxation rates of ^1^H and ^15^N nuclei must be removed. We found that the combination of the Π analysis method (developed by Palmer’s group^11,12^) and the HSQC0 experimental method (developed by Markley’s group^13,14^) offered an effective solution^15^. The determined thermodynamic and kinetic constants differed significantly on a per-residue basis, but the residues in spatial proximity tend to have similar values^15^. Interestingly, we discovered linear relationships in the log k vs. log K and log k′ vs. log K plots^10,15^. In contrast to the conventional LFERs, the data points (log K, log k) and (log K, log k′) in our LFER are derived from different positions in one polypeptide chain. Therefore, we refer to the new type of LFER as residue-based LFER (rbLFER).

Here, we performed a literature search to collect residue-specific equilibrium and kinetic constants of proteins, to determine the applicability of rbLFER in various protein equilibriums with diverse interconversion time scales. A substantial number of the REFER plots exhibited good linearity. Among the successful cases, the HX (hydrogen exchange) study of apomyoglobin folding intermediates is particularly interesting^16^. The majority of the amino acid residues are on a straight line in the REFER plot, but some residues deviate from the line. We found that these outlier residues precisely correspond to the transient translocation of an α-helix in the apomyoglobin folding intermediates, which were first discovered a decade-and-a-half ago by other methods^17,18^. The excellent agreement between the past and present independent analyses demonstrates that rbLFER can reveal the structural and energetic aspects of the dynamic states of protein molecules.

## Results

### Residue-based LFER examples

**Figure 1:**
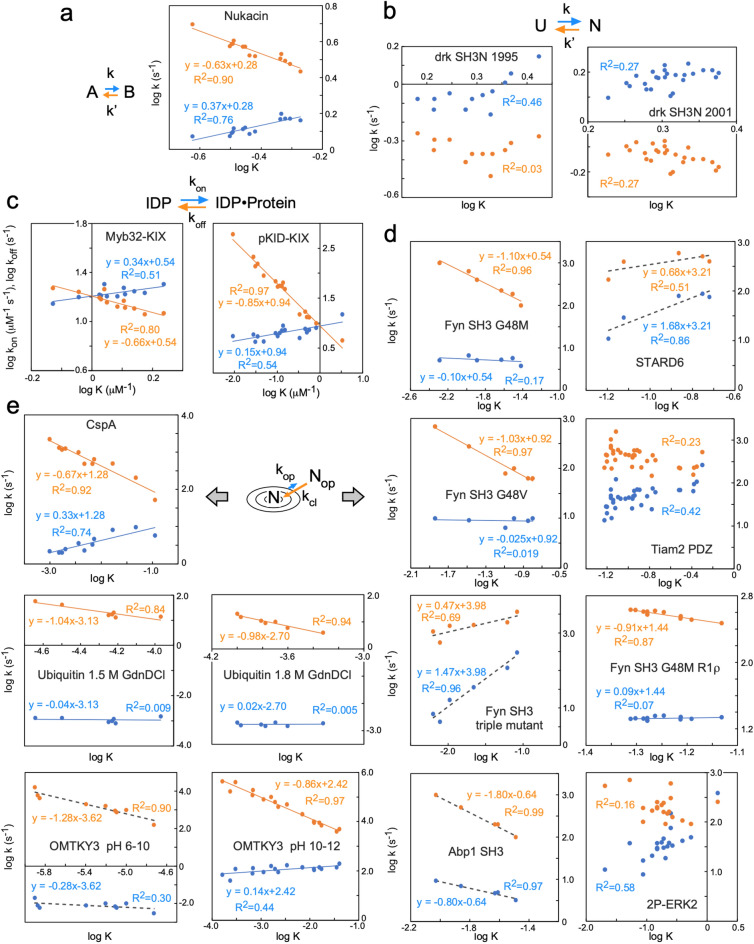
Residue-based REFER plots based on the literature data listed in Table 1. (**a**) Nukacin ISK-1. (**b**) N-terminal SH3 domain from Drosophila drk protein (drk SH3N). The drk SH3N is in an exchange between unstructured state U and native folded state N. There are different data sets from the two reports. (**c**) Myb32 and pKID are intrinsically disordered polypeptides that bind to the KIX domain. (**d**) Structural fluctuations of native states were investigated by the relaxation dispersion (RD) NMR method. The SH3 domains are derived from the Fyn and Abp1 proteins. The mutations in the SH3 domains markedly increased the folding rate despite their destabilization of the folding state, and have suitable properties for the RD studies^34^. The STARD (steroidogenic acute regulatory-related lipid transfer domain) from the STARD6 protein. The PDZ (PSD-95/Dlg/ZO-1) domain from the Tiam2 (T cell lymphoma invasion and metastasis 2) protein. A dual-phosphorylated (2P-ERK2) form of the ERK2 (extracellular signal-regulated kinase 2) protein. (**e**) Structural fluctuations of native states investigated by the HX method. There are two measurement conditions for ubiquitin and OMTKY3 (turkey ovomucoid third domain 3). In (**a**)–(**e**), the least-square lines and data points associated with the forward direction are colored blue, and those associated with the backward direction are orange. The least-square lines with interpretable slopes between 0 and 1 are depicted as solid lines, whereas those with uninterpretable slopes less than 0 or greater than 1 are depicted as dashed lines. No least-square lines are drawn if the correlations are considered insignificant. The concentric circle represents a basin-shaped energy landscape of the structural fluctuations around the native state, N, in equilibrium with the open state, N_op_.

Extensive literature searches led to the collection of reports on residue-specific equilibrium constants and residue-specific kinetic constants of proteins (Table 1). We recovered the data from the tables and figures and generated the corresponding REFER plots (Fig. 1). The techniques used and the phenomena under consideration are quite diverse. The first category, including nukacin ISK-1, is the EXSY NMR analyses of slow exchanges between two sets of cross peaks (Fig. 1a, b)^10,15,19,20^, and the second category is the relaxation dispersion (RD) NMR analyses of the binding of an IDP (intrinsically denatured polypeptide) to a target protein (Fig. 1c)^21,22^. In the case of an IDP, only one set of cross peaks was observed due to the fast exchange between two or more states. Since the state-specific differences in the R_1_ and R_2_ relaxation rates of ^1^H and ^15^N nuclei are averaged out, the information on the exchange process can be extracted from the averaged single cross peaks. The same situation is applied to the structural fluctuations of folded-state proteins studied by RD NMR (Fig. 1d)^23–28^. The HX experiment also provides information on the structural fluctuations of folded proteins (Fig. 1e)^29–32^. Note that the NMR in the HX studies is used for the quantitation of the proton occupancy and does not directly observe the HX phenomena. Indeed, mass spectrometry is applicable in place of NMR^33^. Overall, the majority of the REFER plots showed residue-based linear relationships.

**Table 1:** Proteins used for the generation of REFER plots.

Protein/domain	Conditions of measurement	Method^a^	Assumption	Ref	Source
Nukacin ISK-1	27 res, 3 mono S bonds, 308 K, pH 3.5	^15^N-EXSY		^15^	Figure 3b
Drk SH3N	59 res, 287 K, pH 6.0	^15^N-EXSY		^19^	Figures 3 and 6
	293 K, pH 6.0			^20^	Table 2
Myb32-KIX:MLL28^b^	32 res Myb and 87 res KIX + 28 res MLL, 303 K, pH 7.0	^15^N-RD (CPMG) + titration	Two states	^21^	Figure 5
pKID-KIX	34 res pKID and 87 res KIX, 303 K, pH 7.0	^15^N-RD (CPMG) + titration	Two states^c^	^22^	Table 1
Fyn SH3 G48M	59 res, 288 K, pH 7.0	^15^N-RD (CPMG)	Two states^d^	^23^	Figure 1b
Fyn SH3 G48V	59 res, 283 K, pH 7.0	^15^N-RD (CPMG)	Two states^d^	^23^	Figure 1c
Fyn SH3 mutant	59 res, A39V/N53P/V55L, 308 K, pH 7.0	^15^N-RD (CPMG)	Two states^d^	^24^	Figure 1b
Abl1 SH3 mutant	59 res, 313 K, pH 7.0 E7L/V21K/N23G/G48V	^15^N-RD (CPMG)	Two states^d^	^24^	Figure 1a
STARD6	220 res, apo form, 298 K, pH 7.4	^15^N-RD (CPMG)		^25^	Figure 4
Tiam2 PDZ mutant	90 res, 298 K, pH 6.8 M978L/E979K/F982L/V987L	^15^N-RD (CPMG)		^26^	Table 2
Fyn SH3 G48M	59 res, 298 K, pH 7.0	^15^N-RD (R_1ρ_)	Two states	^27^	Table 1
2P-ERK2 kinase	356 res, dual-phosphorylated form, 298 K, pH 7.4	Methyl ^13^C-RD (CPMG)		^28^	Table 2
Ubiquitin	76 res, 1.5 and 1.8 M guanidine deuterium chloride (GdnDCl), 288 K, pH 7–9	HX/NMR	k_op_ and k_cl_ are independent of pH, EX1 mechanism	^29^	Table 1
CspA	70 res, 311 K, pH 6–11	HX/NMR + NMR (saturation transfer)	k_op_ and k_cl_ are independent of pH, EX1 + EX2 mechanism	^30^	Table 2
OMTKY3	56 res, 303 K, pH 6–10	HX/NMR&ESI–MS	k_op_ and k_cl_ are independent of pH, EX2 mechanism	^31^	Table 1
OMTKY3	56 res, 303 K, pH 10–12	Quenched-flow HX/NMR	k_op_ and k_cl_ are independent of pH, EX1 mechanism	^32^	Table 1
Apomyoglobin	153 res, apo form, ambient temperature, pH 7–11	Quenched-flow HX/NMR	k_op_ and k_cl_ are independent of pH and have identical values at the refolding times of 0.4 and 6 ms. EX1 + EX2 mechanism	^16^	Table 1

^a^Relaxation dispersion (RD) experiments using either the CPMG (Carr–Purcell–Meiboom–Gill) pulse train or a constant radiofrequency spin lock field (R_1ρ_).

^b^The minor binding site for the pKID peptide on the KIX domain was masked by the second IDP peptide derived from the MLL (mixed-lineage leukemia) protein.

^c^In the original report, the authors concluded that the three-state model was appropriate for the coupled binding and folding of the pKID peptide, Free ⇄ Intermediate (encounter complex) ⇄ Bound. Here, the exchange between the free state and the encounter complex was analyzed.

^d^In the original reports, the authors concluded that the three-state model was appropriate for the exchange processes. However, we used the kinetic constants calculated assuming a two-state model to generate the REFER plots.

Here, we discuss the interpretation of the slopes of the REFER plots, by focusing on the forward direction (right-pointing arrows) depicted by the blue lines and their associated data points (Fig. 1). According to a generally accepted interpretation^4,35–37^, the slope represents the structural and energetical similarity between the initial state and the transition state, and hence the value must be between 0 and 1. In the case of structural fluctuations of folded-state proteins (Fig. 1d, e), the blue lines are nearly horizontal, and the slopes are almost zero in many instances. This situation indicates the high similarity of the transition state to the folded state. This is a convincing result, considering that the interactions that stabilize the native folded state must be disrupted at the beginning of the fluctuation. The least-square lines with negative slopes and slopes greater than 1 are difficult to interpret. These cases are depicted by dashed lines. In some cases, particularly for large proteins, the data points are scattered, and no least-square lines are shown. The assumption of the two-state exchange probably does not hold in the case of failure. Understandably, the REFER plots based on the literature data, including those with interpretable slopes, must be properly assessed in the future.

### Alternative representation of residue-based LFER

**Figure 2:**
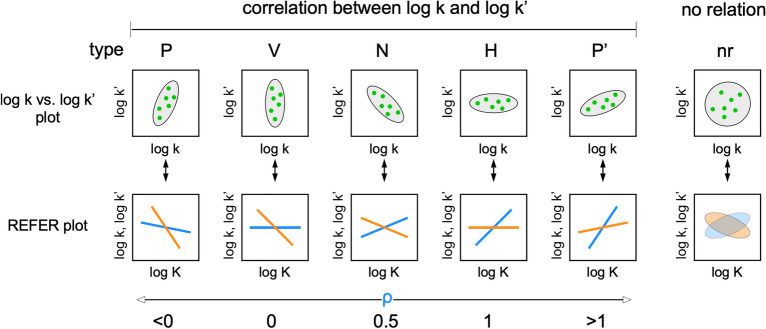
Relationships between log k and log k′ as the basis of rbLFER. Several types are defined according to the data point distributions. Type N shows a negative correlation, and types V and H show flattened distributions of data points with zero correlations. The vertically flattened distribution provides two least-square lines with the slopes ρ of 0 (blue) and − 1 (orange), and the horizontally flattened distribution provides those with the slopes ρ of 0 (orange) and 1 (blue) in the REFER plots. Types P and P′ are positive correlations between log k and log k′. Type nr has no relation between log k and log k′, and the REFER plot may show a weak, artificial correlation trend.

**Figure 3:**
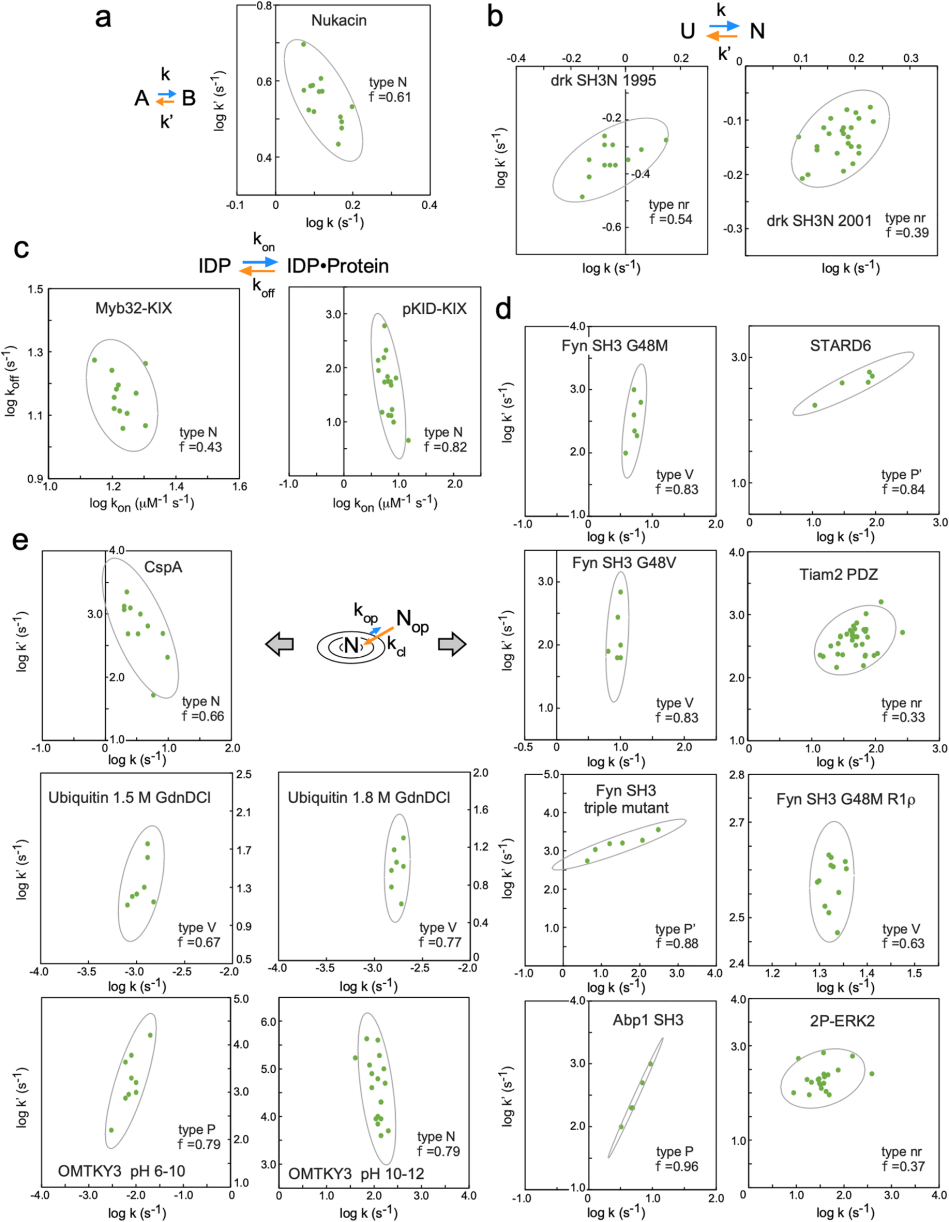
Datapoint distributions in the log k vs. log k′ plots. In each panel, the category type defined in Fig. 2, a 95% confidence ellipse, and the flatness of distribution are shown. The flatness of the distribution is defined by f = 1-b/a, where a is the long axis and b is the short axis of the confidence ellipse. The axis ranges are set equally for the proper interpretation of the flatness of the confidence ellipses.

The physical interpretation of the REFER plot is clear: the two axes, log K and log k, are proportional to the changes in the corresponding free energy terms. However, the two axes are not independent due to the equation, K = k/k′, i.e., log K = log k − log k′, which could generate an artificial linear relationship in the REFER plot (see the next section). To address this issue, we must check a correlation between log k and log k′ for the proper assessment of rbLFER. Figure 2 shows the type classifications of log k vs. log k′ plots, and Fig. 3 shows the log k vs. log k′ plots of the real examples listed in Table 1. The classification types are defined according to the distribution pattern of the data points. Type N is referred to as the negative correlation between log k and log k′, which leads to the blue least-square lines with the slope of 0 < ρ < 1 in the REFER plot (cf. Equation (9) in the previous paper^10^). In extreme cases, when the distribution of data points in the log k vs. log k′ plot has a flattened shape, the least square lines have a slope of 0 or 1 in the REFER plot. According to the orientation of the oval-shaped distribution, vertical and horizontal, their types are defined as V and H, respectively. Since either log k or log k′ is rather constant, the two log terms are uncorrelated in types V and H. Consequently, the Pearson's correlation coefficient R is zero in the log k vs. log k′ plot, and one of the two least-square lines with a zero slope has an almost zero R^2^-value in the REFER plot. This fact indicates that the R and R^2^ values in the two plots are not always good indicators of rbLFER. Instead, 95% confidence ellipses are drawn to quantify the flat distributions (Fig. 3). The closer flatness of the distribution to 1 reflects the higher linearity of the rbLFER.

Next, we consider the case of positive correlations between log k and log k′. If the degree of variation of log k′ is larger than that of log k, the type is P, and if the degree of variation of log k′ is smaller than that of log k, the type is P′. Because the slopes of the blue least-square lines become negative or greater than 1 according to the type, the slopes are not physically meaningful. Such anomalous slope values occur if the exchange process cannot be described by a simple two-state model. For example, the ^15^N relaxation dispersion studies of various mutants of the Fyn SH3 domain revealed the presence of about 1% of the transient intermediate state I in the three-state model, N ⇄ I ⇄ U^23,24,27^. Even though the percentage is small, the assumption of the two-state model is not strictly valid. Another possible cause is unintended measurement biases. The final type is nr (no relation). This is probably due to large measurement errors or accidental problems (see the next section).

### Risk of fake linearity

**Figure 4:**
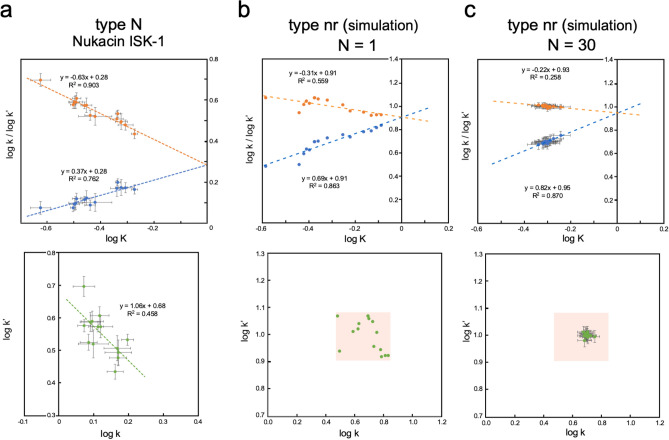
Risk evaluation of linearity overestimation in the REFER plots. (**a**) REFER plot and log k vs. log k′ plot of nukacin ISK-1^15^ as a reference. The error bars represent one standard error. (**b**) REFER plot and log k vs. log k′ plot artificially generated using a synthetic dataset. Assuming a 15-residue protein, 15 k and k′ values are generated as uniformly distributed random numbers between 3 and 7 and between 8 and 12, respectively. The numerical ranges used are shown as the rectangular shape in the log k vs. log k′ plot. (**c**) The random number generation per residue was repeated 30 times (N = 30). The error bars represent one standard error of the mean. Note that one standard deviation is 5.5 (the square root of 30) times larger than one standard error of the mean.

As already mentioned, the triadic relationship among K and two k’s could generate artificial correlations in the REFEP plots. This could lead to misidentification and even doubt about the validity of rbLFER. We start with an extreme example. Fifteen k and 15 k′ values are generated as uniformly distributed random numbers between 3 and 7 and between 8 and 12, respectively. We can choose any other residue number and numerical ranges for the random numbers. Note that single K, k, and k′ values for all residues are a hidden assumption. The log k vs. log k′ plot is type nr, but the REFER plot shows good linearity (Fig. 4b). This is fake, however, caused by the assumption of unnecessarily large measurement errors. This gedankenexperiment tells us the proper control and assessment of measurement errors (i.e., add error bars) is a prerequisite to avoiding fake linearity in the REFER plots. As the number of observations increases, the data points in the REFER plot converged around the averages (Fig. 4c), but some linearities within narrow ranges remain in the REFER plot (although significant enlargement is necessary to recognize them). Thus, simple increase in the number of experimental measurements does not help solve the problem. Instead, it is necessary to simultaneously observe the three correlations between the log K, log k, and log k′ terms to identify a true rbLFER. As a real example, the experimental results of nukacin ISK-1 are shown with error bars (Fig. 4a). The data points corresponding to different residues are well distributed and correlated in the REFER plot and the log k vs. log k′ plot even after repeated NMR measurements (N = 12 for K, and N = 24 for k and k′)^15^.

We recovered the error estimates of residue-specific K, k, and k values (Table 1) and added them in the REFER plots (Supplementary Fig. S1) and the log k vs. log k′ plots (Supplementary Fig. S2). We must treat the error estimates with enough care because of the different definitions (e.g., standard error or standard deviation) and the different methods (e.g., curve fitting error or Monte Carlo estimation). In some cases, the errors appear too large (drk SH3N 2001, STAD6, 2P-ERK2, OMTKY3 pH 6–10, and OMTKY3 pH 10–12), but significant dispersions of log K, log k, and log k′ values are observed in all the cases.

We must also pay attention to measurement biases. The imbalance in the number of experimental values to be obtained and parameters to be determined is a serious problem in the accurate and precise determinations of the residue-specific equilibrium and residue-specific rate constants. As for ^1^H–^15^N NMR, the resonance- and state-specific NMR parameters, such as the relaxation rates, R_1_ and R_2_, of ^1^H and ^15^N nuclei must be considered. Global fitting is a solution, but only the average value over many residues is available. Alternatively, an appropriate assumption can be introduced to reduce the number of fitting parameters. For example, some parameters are supposed to remain unchanged under different measurement conditions (Table 1). Therefore, we must pay attention to the risk that unexpected adverse effects caused by obligatory assumptions could generate an artificial linear relationship in REFER plots. In fact, too good correlations in the log k vs. log k′ plots are questionable in the Fyn SH3 triple mutant and Abp1 SH3 cases (Supplementary Fig. S2). However, it is unreasonable to attribute all LFERs in Fig. 1 to measurement biases because a wide variety of methods were used.

In summary, the influences of measurement-specific biases and measurement errors must be considered seriously. The moderate linearity in the REFER plots does not simply prove a direct connection between the equilibrium constants and rate constants. The correlations in the log k vs. log k′ plot must be examined to avoid such a misinterpretation (Fig. 3; Supplementary S2). In this context, as independent evidence, a special insight revealed by the REFER plot can be tested by the results obtained from other experiments. The retrospective analysis of apomyoglobin folding intermediates in the next section provides a clear illustration of this point.

### HX experiment of apomyoglobin

Information on the structural fluctuations of proteins can be obtained by monitoring the hydrogen/deuterium exchange of backbone amide protons with bulk water. The exchange mechanism consists of two processes: a two-state exchange of structural conversion and the exchange of isotopes^38^.

1 $$NH \left( {closed} \right) \begin{array}{*{20}c} {\to ^{{k_{op} }} } \\ {\mathop \leftarrow \limits_{{k_{cl} }} } \\ \end{array} NH\left( {open} \right)\to ^{{k_{{\text{int}}} }} ND\left( {exchanged} \right)$$

where NH(closed) is a folded state in which amide protons are protected from the exchange, and NH(open) is an open state in which exchange occurs. The H/D exchange rate, k_int_, is highly dependent on the pH of the solution. Usually, a single high pH pulse is used in the EX1 regime (k_int_ ≫ k_cl_) to simplify the analysis. Alternatively, a wide pH range of labeling pulses can be used. Since the pH dependence of k_int_ is well known, k_op_ and k_cl_ can be determined without assuming the exchange mechanism, either EX1 (k_int_ ≫ k_cl_) or EX2 (k_int_ ≪ k_cl_). After a hydrogen/deuterium exchange reaction, NMR is used to measure the proton occupancy of each residue in an acidic solution. Mass spectrometry is also used after protein fragmentation by protease digestion.

**Figure 5:**
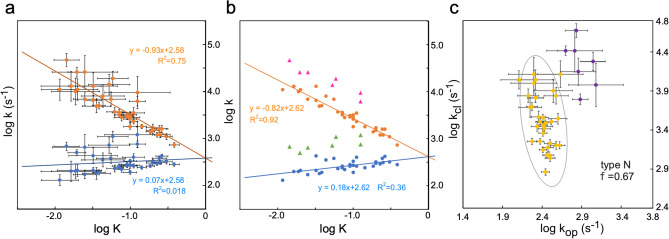
REFER plot and log k vs. log k′ plot of apomyoglobin folding intermediates. (**a**) REFER plot using all observed residues with estimated fitting uncertainties^16^. The data points and least square lines of the log k_op_ vs. log K plot and the log k_cl_ vs. log K plot are blue and orange, respectively. (**b**) Replot. Outlier residues (green and magenta) were removed to redraw the least-square lines (blue and orange). See Supplementary Fig. S4 for details. (**c**) Log k vs. log k′ plot. The yellow dots are the residues contributing to the least-square lines, and the purple dots are the outlier residues in (**b**). The yellow group belongs to type N. The yellow dots are enclosed by a 95% confidence ellipse.

Sperm whale myoglobin is a popular model protein for understanding protein folding^39,40^. Myoglobin is a globular protein consisting of eight α-helices, designated A to H. The apo form of the protein has almost the same structure as the heme-bound holo form. Within the initial burst phase of apomyoglobin refolding, two kinetic intermediates, designated as I_a_ and I_b_, are sequentially formed^41^. In the state I_a_ structure, the major portions of helices A, G, and H and part of helix B are established. Subsequently, parts of helices C, D, and E are formed and added to the already-existing helices in the state I_b_ structure^39,40^. Wright's group performed quenched-flow hydrogen exchange experiments, using a continuous-flow mixer, to determine the residue-specific kinetic parameters, k_op_ and k_cl_, of the folding intermediates^16^. The k_op_ and k_cl_ values were each assumed to remain unchanged in the two labeling pulse durations of 0.4–4.0 ms and 6.0–9.6 ms, and simultaneous numerical fitting was performed to obtain a more accurate estimation of the rate constants. Consequently, the rate constants are averaged values of I_a_ (0.4–4.0 ms) and I_b_ (6.0–9.6 ms). We collected the k_op_ and k_cl_ data and associated errors from the literature^16^ and constructed the REFER plot. We found that the (log K, log k_op_) and (log K, log k_cl_) data points were modestly aligned around straight lines (Fig. 5a). Then, we performed a robust linear regression to iteratively calculate the weight of each data point. Outlier residues were identified by robust regression as a data point with a small weight value (Supplementary Fig. S3) in a statistically objective manner. Seven outlier residues (A134, L135, E136, R139, D141, I142, and A143) were found and removed (green and magenta, Fig. 5b) to redraw the least-square lines. Due to the large measurement errors (Fig. 5a), the exceptional handling may not be convincing in the revised REFER plot (Fig. 5b), but the outlier residues are outside of the 95% confidence ellipse in the log k vs. log k′ plot (Fig. 5c).

**Figure 6:**
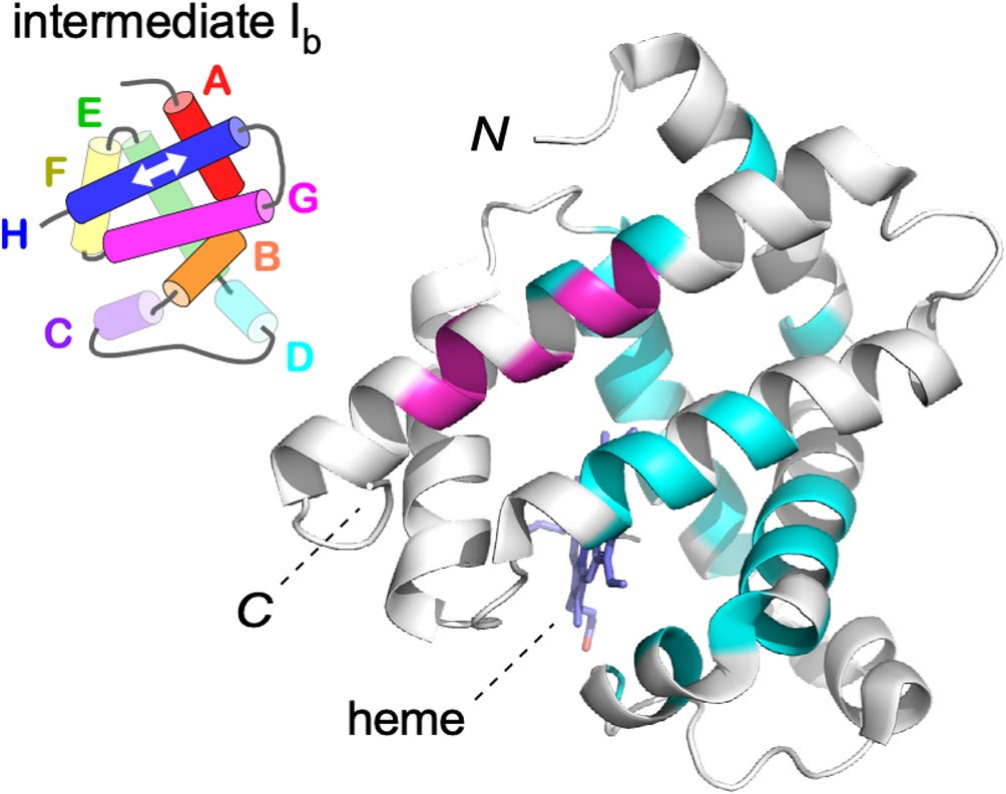
Mapping of the outlier residues in the REFER plot on the N-state structure of myoglobin. The native holo-structure (PDB ID 2JHO) is used as the best alternative to the intermediate structures. The residues on the least-square lines in Fig. 5b are colored cyan and the outlier residues are magenta. The other residues without information are colored white. These unprobed residues include parts of helices A, G, and H due to the full protection of the amide protons, and helix F and the loops due to the lack of local secondary structures in the intermediate states. The inset shows the helical topology of the kinetic intermediate I_b_. The major portions of helices A, B, G, and H are formed. The half-translucent cylinders represent partially formed helices C, D, and E. The state of helix F is unclear in the folding intermediate states because helix F is not stably packed in the apo state of apomyoglobin. The white arrow indicates the translocation of helix H in the intermediate states, I_b_.

The outlier residues were mapped on the native-state structure of apomyoglobin. All outlier residues are located on one face of helix H (Fig. 6, magenta). Interestingly, the Wright group showed that the intermediate I_b_ is a mixed state of two conformations, with one containing the translocation of helix H by one helical turn toward its N-terminus relative to helix G (Fig. 6, *inset*). They drew this conclusion from the HX study of amino acid mutants^17^ and the combination of the HX study with fluorescence quenching and FRET (Förster resonance energy transfer) measurements^18^. The translocated helix H is not present in the folded state N, and thus they referred to the translocated form of helix H as a non-native structure. Strikingly, the region highlighted by the outlier residues precisely coincides with the amino acid residues involved in the helix translocation.

## Discussion

Our retrospective analysis showed that the linear relationship between residue-specific log K (the logarithm of the equilibrium constant) and residue-specific log k (the logarithm of the rate constant) in the REFER plot holds for the structural changes of many proteins (Fig. 1). The analytical methods include EXSY NMR, dispersion relaxation NMR, and hydrogen/deuterium exchange measurements. The residue-based LFER is seen in the two-state slow exchange (Fig. 1a) and structural fluctuations (Fig. 1d, e) of monomeric small proteins, and in two-molecule systems, such as IDP-protein interactions (Fig. 1c). Disappointingly, rbLFER is not seen in the dynamic equilibrium between the unfolded (U) and folded (N) states of the drk SH3 domain (Fig. 1b). In the original report, specially designed pulse sequences were used for the cancellation of the different relaxation properties of magnetization associated with the U and N states^20^, but the correction is only valid for the reverse INEPT step and seems insufficient.

The substantial number of rbLFER instances indicates the applicability of rbLFER to a wide variety of protein-related phenomena. In this context, the inadvertent use of “two-state exchange” is potentially confusing. Traditionally, “two-state exchange” is used for systems exhibiting the property of cooperativity. Due to the assumption of ideal perfect cooperativity among residues, single K, k, and k′ values suffice for the description of the two-state exchange from a macroscopic standpoint. Under the rbLFER concept, however, the K, k, and k′ values are different on a per-residue basis, and in other words, one macroscopic state looks different from one residue to another. We propose the use of “two-state exchange with reduced cooperativity” as a near-term solution to distinguish from the traditional “two-state exchange”. We expect that the “two-state exchange” will naturally include the residue-level heterogeneity, with an increased interest in the rbLFER concept in the future.

The linearity of the REFER plot is a measure of the deviation from the ideal smooth structural changes. In particular, the reanalysis of the HX study of apomyoglobin^16^ is intriguing. We found that the outlier residues deviated from the least-square lines in the REFER plot of the apomyoglobin folding intermediates (Fig. 5). The distribution of the outlier residues in the three-dimensional structure is in good agreement with the transient translocation of helix H in the intermediate state I_b_ (Fig. 6). This unexpected outcome demonstrates that the rbLFER is a practical method to study the dynamic aspects of proteins. The outlier data points appear to form second lines that are almost parallel to the first least-square lines (Fig. 5b; Supplementary Fig. S4b), which suggest a collective motion of the outlier residues. The transient translocation of helix H detected in other experiments is a suitable mechanism for collective motion. The hydrogen bond breaks caused by the translocation of helix H accelerate the hydrogen exchange rates of amide protons. The rate increase was about 1000 s^−1^, considering the rise in the intercept values (Supplementary Fig. S4b). Note that the REFER plot (Fig. 5) is about the structural fluctuations of the apomyoglobin folding intermediate I_b_, whereas the translocation of helix H (Fig. 6) was detected in the folding intermediate state during the entire folding process of apomyoglobin. The two phenomena must be closely related, but further theoretical and experimental studies are necessary.

The rbLFER indicates that the per-residue thermodynamic and kinetic energy terms are closely related throughout a polypeptide chain. We suggest that the rbLFER is a physicochemical basis for smooth folding and conformational changes of protein molecules. In application, the rbLFER provides a useful tool for studying the structures and energetics of the dynamic states (in particular, the transition states) of protein molecules.

## Methods

We performed a literature search to collect residue-specific equilibrium and residue-specific rate constants of proteins, mainly in the PubMed literature database (http://www.ncbi.nlm.nih.gov/pubmed/). The keywords included ‘two states’, ‘two sets of cross peaks’, ‘exchange spectroscopy’, ‘residue-specific’, LFER, etc., and their combinations. The linear regression analyses of the REFER plots and log k vs. log k′ plots were performed in the Excel files. To identify outlier data points in REFER plots, robust regression was performed in MATLAB R2020b using the ‘fitlm’ command with the ‘RobustOpts’ option. The Excel files and the MATLAB source code are available as Supplementary Datasets S1 to S5. The protein cartoon was generated with the program PyMOL, version 2.4.2 (Schrödinger). The cartoon image of the apomyoglobin was generated using the PDB ID 2JHO.

## Supplementary Information

Supplementary Information 1.

Supplementary Information 2.

Supplementary Information 3.

Supplementary Information 4.

Supplementary Information 5.

Supplementary Information 6.

## Data Availability

All data needed to evaluate the conclusions in the paper are presented in the paper and the supplementary information.
