# Supplementary material for: Retrospective study for the universal applicability of the residue-based linear free energy relationship in the two-state exchange of protein molecules
Source: Sci Rep. 2022 Oct 7;12:16843. doi: 10.1038/s41598-022-21226-z (PMC9546931; doi:10.1038/s41598-022-21226-z)
Supplement: Supplementary file 6 — Supplementary Information 6. [file 41598_2022_21226_MOESM6_ESM.docx]

Supplementary Information

**Retrospective study for the universal applicability of the residue-based linear free energy relationship in the two-state exchange of protein molecules**

Daisuke Fujinami, Seiichiro Hayashi, and Daisuke Kohda*

Medical Institute of Bioregulation, Kyushu University, Fukuoka 812-8582, Japan

Correspondence to Daisuke Kohda, E-mail: [kohda@bioreg.kyushu-u.ac.jp](file:///G:\nyudata\kohda@bioreg.kyushu-u.ac.jp)

**This PDF file includes:**

Legends for Datasets S1 to S5

Figures S1 to S4

**Legends for Datasets S1 to S4**

**Dataset S1 (separate file).** Excel file to generate the log k vs. log K plots (Source data for Figs. 1 and S1)

**Dataset S2 (separate file).** Excel file to generate the log k vs. log k’ plots (Source data for Figs. 3 and S2)

**Dataset S3 (separate file).** Excel file to generate the log k vs. log K plots and log k vs. log k’ plots using the artificially generated datasets (Source data for Fig. 4)

**Dataset S4 (separate file).** Excel file to generate the two types of plots of the apomyoglobin folding intermediates (Source data for Fig. 5)

**Dataset S5 (separate file).** MATLAB m-file to perform the iteratively reweighted least squares regression


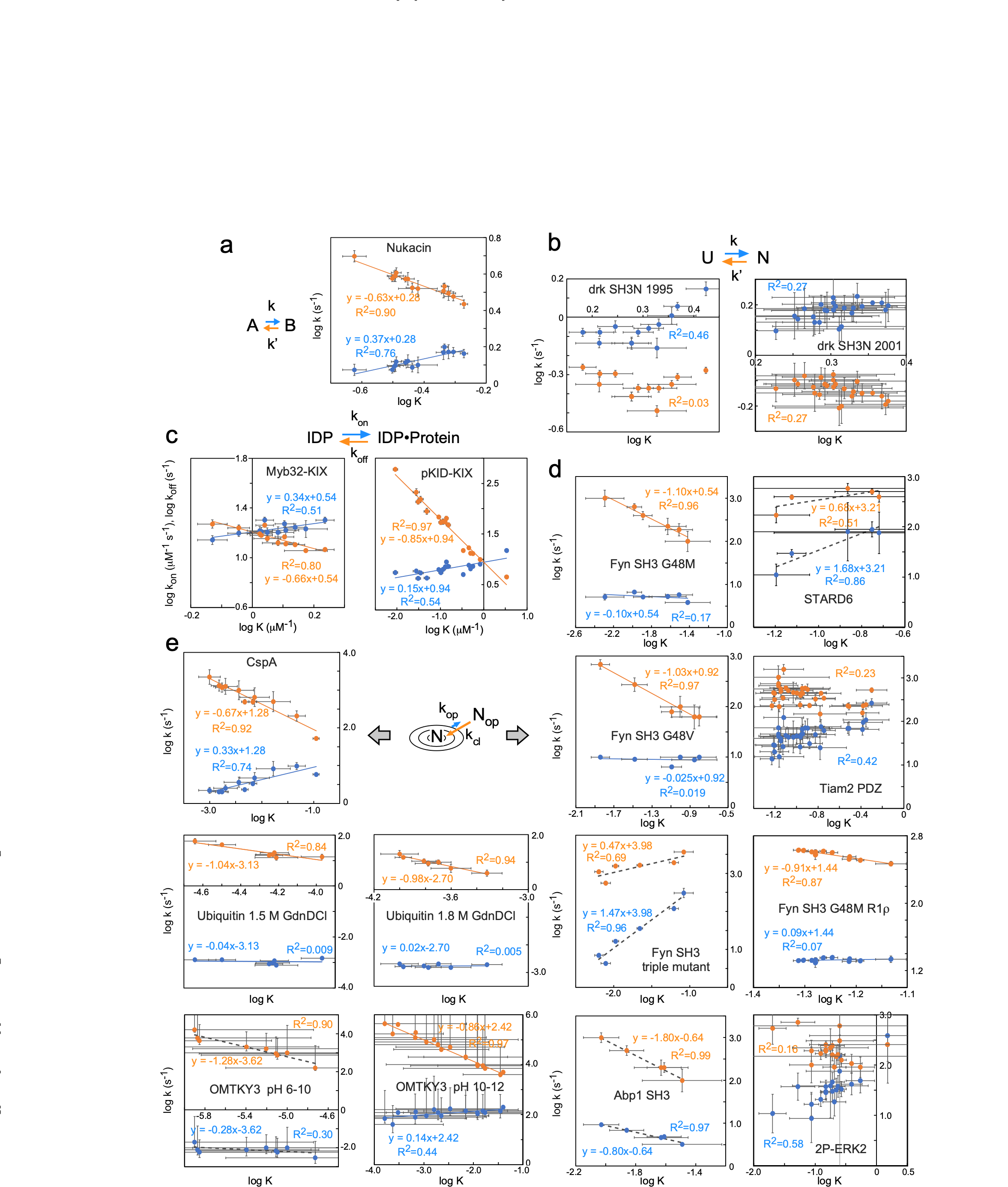


**Figure S1.** The same as Fig. 1 but with error bars.


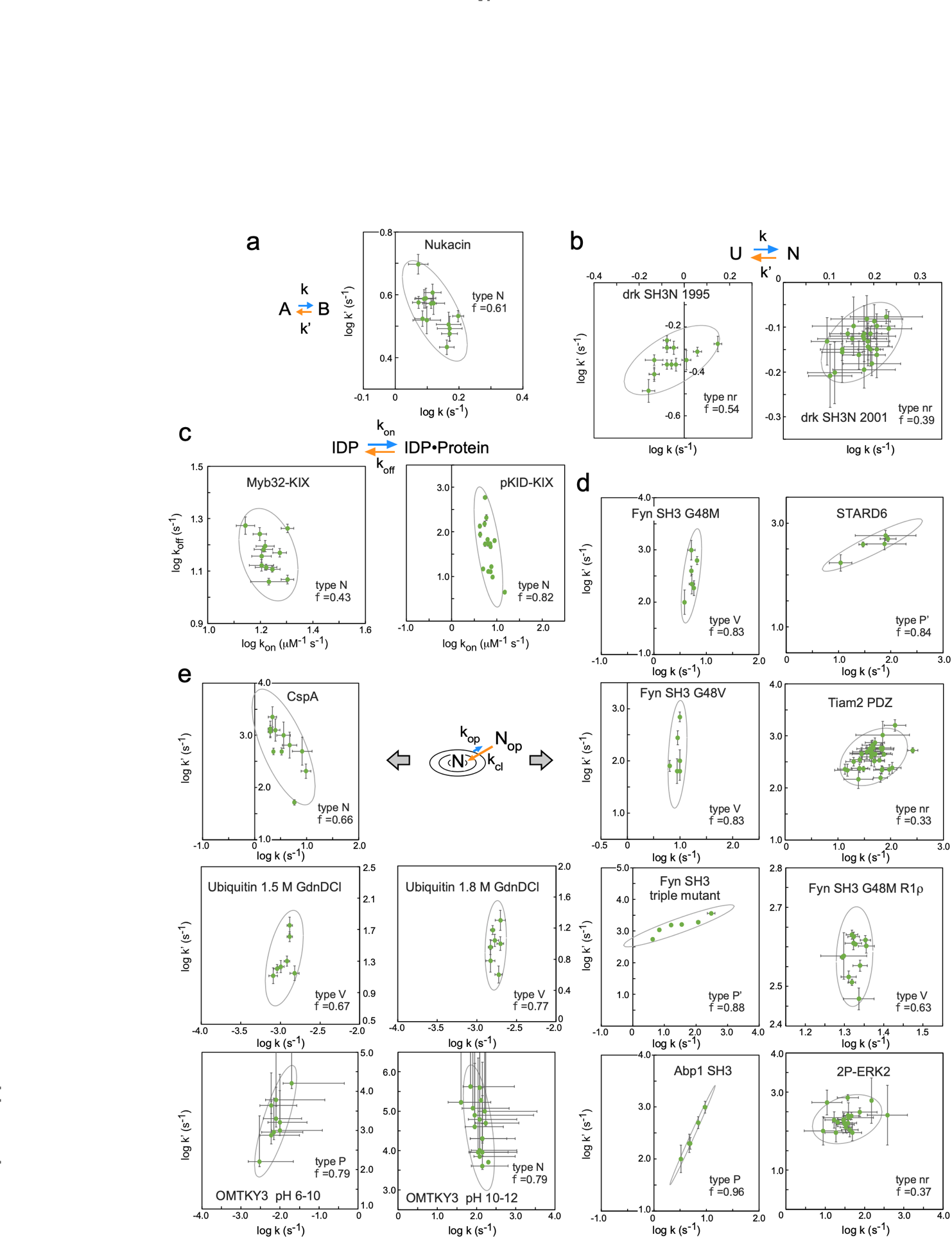


**Figure S2.** The same as Fig. 3 but with error bars.


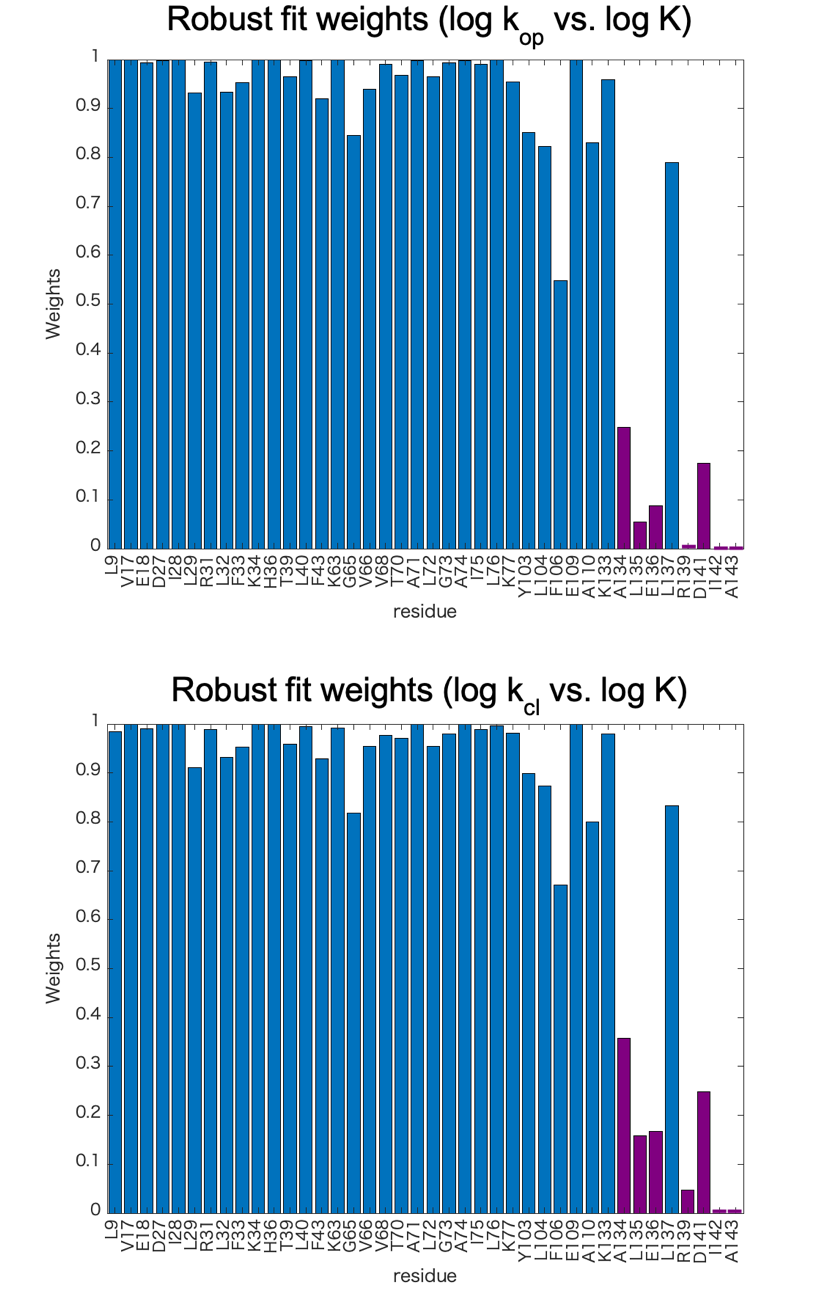


**Figure S3.** Robust fit weights as a function of residue number. Outlier residues (purple bars) in the REFER plot of the HX study of apomyoglobin folding intermediates were identified as data points with small weight values in the iteratively reweighted least squares method.


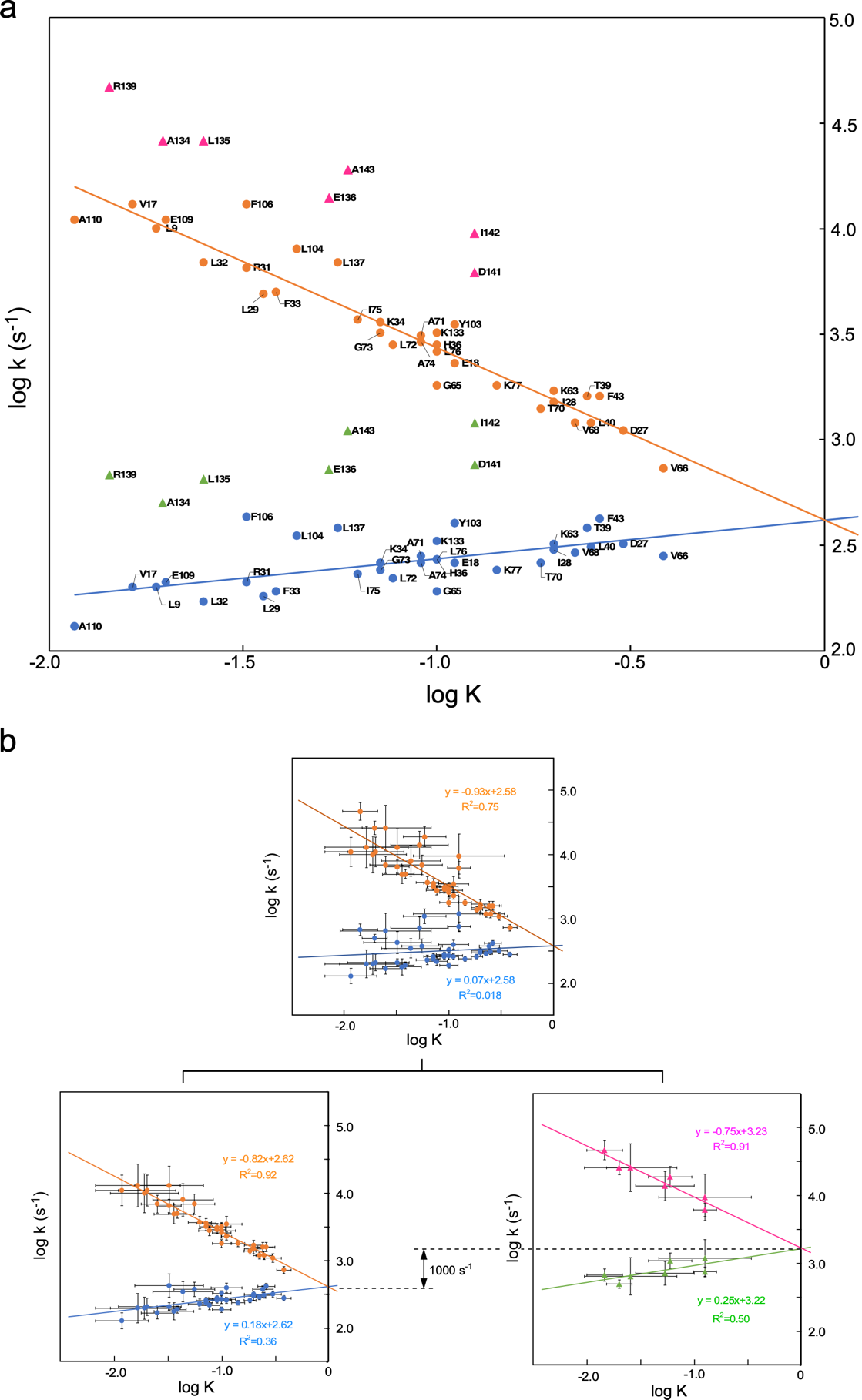


**Figure S4.** Details of the REFER plot of the apomyoglobin folding intermediate. (**a**) Labeling of amino acid residues. (**b**) Error bars of the first least-square lines (blue and orange) and the second least-square lines (green and magenta).
